# Frova Introducer in Anesthesiology: Friend or Foe?

**DOI:** 10.7759/cureus.90723

**Published:** 2025-08-22

**Authors:** Diogo Morais, Armando Cordeiro, Sara Rêgo

**Affiliations:** 1 Anesthesiology, Unidade Local de Saúde de Trás-os-Montes e Alto Douro (ULSTMAD), Vila Real, PRT; 2 Anesthesiology, Unidade Local de Saúde do Nordeste, Bragança, PRT

**Keywords:** airway exchange catheter, frova introducer, post-extubation complications, substernal goiter, tracheal injury

## Abstract

The Frova Intubating Introducer is widely used in airway management, although it carries risks such as barotrauma and airway injury, especially when over-inserted (>26cm) or used for oxygen delivery (>2 L/min). We report a case of a 70-year-old woman with a substernal goiter and chronic obstructive pulmonary disease who developed bilateral pneumothorax, pneumomediastinum, and subcutaneous emphysema after open thyroidectomy and extubation over a Frova catheter. Emergent reintubation and chest drainage were required. Although a tracheal tear was suspected, it was not confirmed. The patient recovered fully with conservative treatment. Our report highlights the need for caution when using airway exchange catheters in patients with challenging anatomical airways and close post-extubation monitoring.

## Introduction

The Frova Intubating Introducer (Cook Medical, Bjaeverskov, Denmark) is a commonly used device in airway management, particularly in situations involving difficult intubation, tube exchange, or extubation of high-risk patients. Designed with a length of 70 cm and an outer diameter of 4.6 mm, it facilitates tracheal tube placement by acting as a flexible guide through the glottis. Due to its effectiveness and ease of use, the Frova has become a standard tool in the management of challenging airways [1,2]. The 2015 guidelines from the Difficult Airway Society (DAS) support the use of airway exchange catheters (AECs) such as the Frova for extubation in patients with risk factors such as difficult airway anatomy, airway edema, or limited respiratory reserve, as these devices maintain access to the airway should reintubation be required [3].

However, despite its clinical utility, the Frova is associated with potentially serious complications, including mucosal trauma, vocal cord injury, subglottic lacerations, barotrauma, and pneumothorax, particularly when the introducer is advanced beyond 26 cm or when resistance is encountered during insertion [4-5]. The administration of oxygen through the Frova has also been implicated in cases of barotrauma and tension pneumothorax. In a porcine model, when AECs were inserted beyond 26 cm, even flows of oxygen ranging from 2 to 4 L/min caused barotrauma in a matter of seconds [6]. Potential causal mechanisms range from high airway pressures to restriction of the gas outflow pathway [6]. Furthermore, the manufacturer's instructions for the use of Frova for AECs do not document a recommended safe flow for the delivery of oxygen. A study of the use of the Frova single-use introducer in 200 patients reported an incidence of airway trauma, defined as the presence of blood on pharyngeal and tracheal suction, of ~5% [7].

We present a case of a patient with a substernal goiter who developed life-threatening complications including bilateral pneumothorax, pneumomediastinum, pneumoperitoneum, and subcutaneous emphysema following elective extubation over a Frova catheter.

## Case presentation

**Figure 1:**
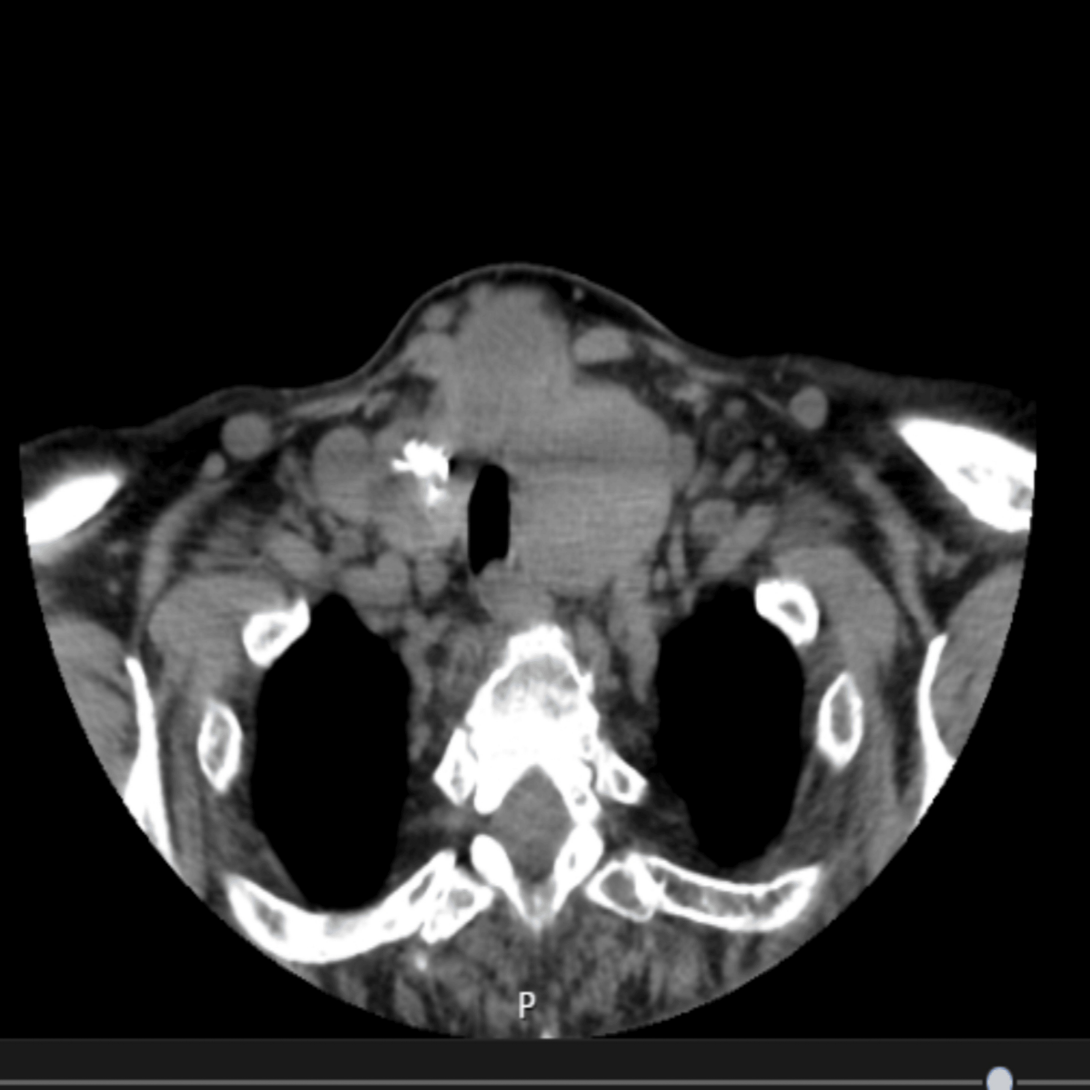
Thoracic computed tomography exhibiting a substernal goiter with tracheal deviation

A 70-year-old female was scheduled for open thyroidectomy due to a substernal goiter. Her past medical history included atrial fibrillation on anticoagulation, arterial hypertension, chronic obstructive pulmonary disease, and obstructive sleep apnea requiring continuous positive airway pressure and oxygen therapy (2 L/min) at night. Current medications included dapagliflozin 10 mg, metformin 1,000 mg, rivaroxaban 20 mg, digoxin 0.125 mg, bisoprolol 5 mg, furosemide 40 mg, and bronchodilators. She had previously undergone mitral valve repair via open heart surgery. Airway evaluation revealed no signs suggestive of a difficult airway: the trachea was midline, and there were no compressive symptoms such as dysphagia, dysphonia, or dyspnea. Preoperative cervical and thoracic computed tomography (CT) confirmed an enlarged thyroid with minimal rightward tracheal deviation as well as minimal anterior wall compression (Figure 1).

Induction of anesthesia was uneventful. Intubation was achieved on the first attempt using videolaryngoscopy with a 6.0-mm reinforced endotracheal tube. During the surgery, a total intravenous anesthesia with propofol was used. The American Society of Anesthesiologists monitoring standards were used, along with bispectral index, quantitative neuromuscular monitoring, and continuous central temperature assessment. The patient was ventilated with lung-protective parameters. The surgery lasted 90 minutes and was uneventful.

**Figure 2:**
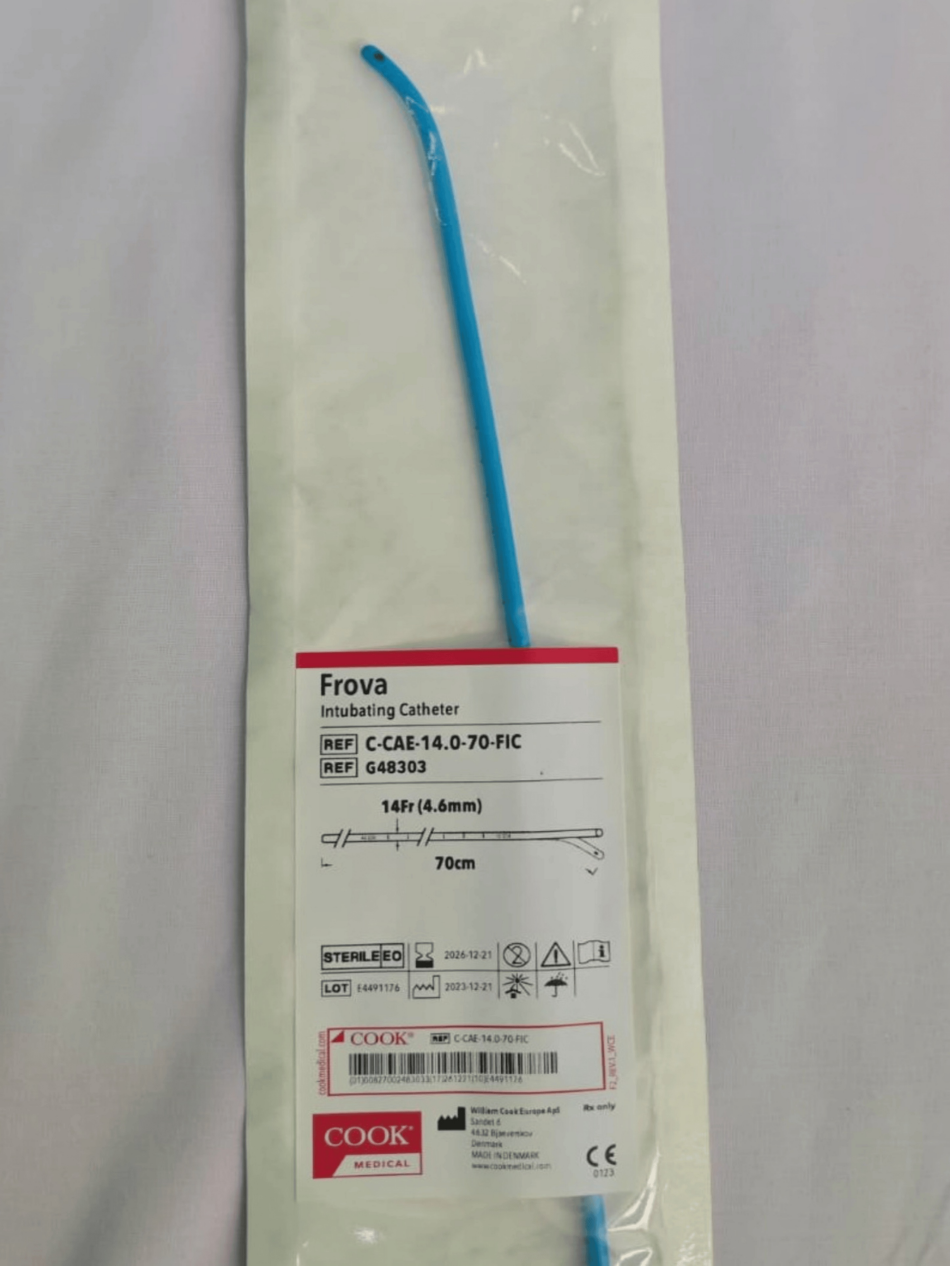
Frova intubating catheter

At the conclusion of the procedure, neuromuscular blockade was reversed with 200 mg of sugammadex. After confirming adequate spontaneous ventilation, a train-of-four ratio >90%, corneal reflex, and spontaneous breathing, the patient was extubated over a Frova over-inserted to 35 cm, with supplemental oxygen at 3 L/min via the catheter (Figure 2). Shortly after extubation, strenuous cough accompanied by subcutaneous emphysema of the face, neck, and upper limbs, and exuberant tongue edema developed rapidly. Emergent reintubation was performed via videolaryngoscopy after Frova removal on the first attempt. Mechanical ventilation revealed high peak (40 cm H₂O) and plateau (30 cm H₂O) pressures, with reduced breath sounds on the left. The patient developed profound bradycardia and hemodynamic instability, requiring 1 mg of atropine and 100-mcg epinephrine bolus. A left-sided chest tube was placed, resulting in immediate improvement in both ventilation and hemodynamics. Arterial blood gas analysis revealed metabolic acidosis and elevated lactate. The values are reported in Table 1.

**Table 1:** Arterial blood gas analysis results

Parameter	Value	Reference values
pH	7.1	7.35-7.45
pCO_2_	67 mmHg	35-45 mmHg
HCO₃⁻	11 mmol/L	22-28 mmol/L
Lactate	6.6 mmol/L	<1 mmol/L
Hemoglobin	12 g/dL	12-16 g/dL

An ultrasound-guided brachial arterial line and subclavian central venous access were established. Urgent CT confirmed the extensive subcutaneous emphysema involving the face, cervical region, and both upper limbs, as well as a bilateral pneumothorax, pneumomediastinum, and pneumoperitoneum. A right-sided chest tube was then established, and a bronchofibroscopy suggested a 1- to 2-cm longitudinal tracheal tear immediately distal to the vocal cords. The patient was transferred to a tertiary center with thoracic surgery capability. Twelve hours after the critical incident, bronchoscopy did not confirm a full-thickness tracheal injury, and surgical intervention was deemed unnecessary.

Afterwards, in the intensive care, on mechanical weaning, left hemiplegia was noted. Cerebral CT showed no signs of hemorrhage or ischemia. Intravenous thrombolysis was considered unsuitable, and a conservative management strategy was adopted. The patient was successfully extubated within 24 hours, as neurological deficits as well as cutaneous emphysema dramatically improved. She then returned to our hospital, first to intensive care, where she stayed for another 24 hours, and afterwards to the surgical ward. Prior to ward transfer, the patient was hemodynamical compensated, with improvement in neurological deficits, as well as subcutaneous emphysema, pneumomediastinum, and pneumoperitoneum. Chest tubes were removed on day 4. Upon hospital discharge, 11 days after the surgery, the patient had fully recovered from emphysema and pulmonary complications, with only minor residual deficits - mild dysarthria and minor left lower limb weakness (grade 4/5 muscle strength).

## Discussion

The Frova Intubating Introducer is a valuable adjunct in managing difficult airways, facilitating endotracheal tube placement, tube exchange, and safe extubation in high-risk patients. Its clinical utility is well-established, particularly in preserving airway access post-extubation. However, despite its benefits, complications associated with its use remain a significant concern.

Rare but severe complications such as pneumothorax, pneumomediastinum, pneumoperitoneum, and subcutaneous emphysema can occur following elective extubation over a Frova catheter. Several mechanisms could account for the complications observed. First, the insertion depth of 35 cm significantly exceeded the consensus-based recommendation safe limit of 26 cm, potentially increasing the risk of airway perforation, particularly in patients with altered tracheal anatomy or underlying pathology [3]. Second, vigorous coughing following extubation may have contributed to airway trauma or exacerbated a pre-existing mucosal injury. Third, oxygen administration even at modest flow rates (>2 L/min) has been implicated in barotrauma and pneumothorax, and its safety remains controversial in the literature [5,6]. Although initial bronchoscopy suggested a tracheal tear, the injury was not confirmed. This discrepancy may reflect a mucosal injury or transient mucosal disruption rather than a complete laceration. The patient’s favorable response to conservative management supports our claim. Importantly, surgical factors may have contributed to the patient’s susceptibility to airway injury. Thyroidectomy, especially in the context of a substernal goiter, can involve tracheal manipulation, potentially weakening it or causing local edema. Additionally, neck hyperextension during thyroid surgery and inflammation of peritracheal tissues may alter the tracheal geometry, making it more vulnerable to trauma from rigid introducers. Although the intraoperative course was uneventful, the possibility of iatrogenic pleural laceration in a patient with preoperative cardiac surgery cannot be excluded as a major trigger to the cascade of complications.

A balance between securing an airway and avoiding iatrogenic harm in vulnerable patients is critical. While extubation over a Frova is endorsed by DAS for high-risk extubations, its use requires careful consideration of patient-specific risk factors. Furthermore, a vigilant post-extubation monitoring, particularly when using adjuncts such as AECs, is of paramount importance. Prompt recognition of complications, as demonstrated by the swift reintubation and pneumothorax decompression, is critical for patient outcomes.

## Conclusions

In conclusion, while the Frova Introducer remains a valuable tool in the anesthesiologist’s armamentarium, its safe use needs to take into account the nuanced understanding of patient anatomy, surgical context, and potential complications. We advocate for enhanced vigilance, strict adherence to safety guidelines, and heightened caution when extubating high-risk patients using AECs. In high-risk extubations, the Frova should be inserted ≤26 cm, oxygen insufflation should be minimized, and close monitoring should be ensured for the early detection of barotrauma.
